# Milk Protein Polymer and Its Application in Environmentally Safe Adhesives

**DOI:** 10.3390/polym8090324

**Published:** 2016-08-31

**Authors:** Mingruo Guo, Guorong Wang

**Affiliations:** 1 College of Food Science, Northeast Agricultural University, Harbin 150030, China; 2 Department of Nutrition and Food Sciences, The University of Vermont, Burlington, VT 05405, USA; guorong_wang@yahoo.com

**Keywords:** milk protein, casein, whey, glue, non-food, polymer

## Abstract

Milk proteins (caseins and whey proteins) are important protein sources for human nutrition; in addition, they possess important natural polymers. These protein molecules can be modified by physical, chemical, and/or enzymatic means. Casein is one of the oldest natural polymers, used for adhesives, dating back to thousands years ago. Research on milk-protein-based adhesives is still ongoing. This article deals with the chemistry and structure of milk protein polymers, and examples of uses in environmentally-safe adhesives. These are promising routes in the exploration of the broad application of milk proteins.

## 1. Introduction

Bovine milk contains about 3.2% protein, 3.4% fat, and 4.8% lactose, as well as various minerals and vitamins. It is an important food for most people around the world. Due to its high nutritional value, milk and its components are widely used in foods in various forms. Additionally, non-food applications are also a large part of milk utilization, dating back to the ancient Egyptians in their use of casein as glue [1] till today’s applications, such as in environmentally-safe wood adhesives and paper/label adhesives [2,3,4].

Milk proteins are categorized as caseins and whey proteins, based on different solubilities at a pH of 4.6 [5], which are approx. 2.6% and 0.6%, respectively, in bovine milk. The properties of casein and whey protein (such as the chemistry, structure, functionality, nutrition, etc.) have been extensively studied over the past few centuries. Casein exists in fresh milk in the form of a “micelle” structure, which is a complex aggregate of proteins (α-, β-, and κ-casein) and colloidal phosphate calcium (CCP) [6]. Whey proteins are a group of globular proteins, which consist mainly of β-lactoglobulin (β-Lg), α-lactalbumin (α-La), and bovine serum albumin (BSA). Both caseins and whey proteins exhibit unique polymer properties [7,8,9].

As with many other naturally-produced polymers, such as starch, tree gums, and clays, milk protein components exhibit excellent adhesive properties, and have been used as one of the major natural adhesive ingredients for thousands years, until the advent synthetic petroleum-based polymers [10]. Natural adhesives are safe, renewable, and environmentally safe; in addition, they possess excellent bonding properties. One of the reasons why caseins or natural polymers have been steadily replaced by synthetic polymers is the cost [2], as milk protein is a food of high nutritional value, and the demands for milk protein for use in food has been increasing [10]. However, the concepts of natural and environmentally-safe adhesives are gaining more and more public attentions. Events where children eat glue/paste, or where people get sick from formaldehyde emissions, have been reported. Natural and safe polymers should be reconsidered for use as adhesives in situations such as office and school paper glues, wood adhesives, and any other high-value-added adhesives.

Due to the development of modern technology, the chemistry, structure, and functionality of milk proteins have been extensively studied. This new knowledge is helpful in the formulation of more efficient and safe adhesives. The objective of this article is to review and discuss the application of milk protein in the formulations of environmentally-safe adhesives, and summarize current and past examples of milk-protein-based adhesives.

## 2. Physicochemical Properties of Milk Proteins

Milk proteins are a heterogeneous mixture of proteins, and constitute approximately 3.0%–3.5% of bovine milk. About 80% of bovine milk protein is called casein, which precipitates at pH 4.6. The protein remains that are soluble in milk serum at pH 4.6 are called whey protein. The typical compositions of milk proteins are listed in Table 1. In general, casein and whey proteins have very different properties. Casein is mostly a random coil with a high proline content, highly phosphorylated for calcium binding, and exists in large casein micelles; whey protein has ordered secondary structures with a low proline content, is non-phosphorylated, and has small soluble proteins. In addition, casein is sensitive to acid and is stable when heated, while whey protein is stable in acid and is sensitive to heat.

### 2.1. Caseins

Due to the high level of proline residue, casein molecules are able to form α-helix and β-sheet, which are essential factors for secondary protein structures [11]. About 85% to 90% of casein in bovine milk exists in a colloidal form, known as micelles [12,13], which are porous, spherical aggregates with diameters ranging from 50 to 600 nm [14,15]. Various models of casein structure have been suggested [6,9,12,14,16,17]. It has been suggested that the integrity of the micelle is attributed to the hydrophobic interactions between casein molecules [18], between caseins and calcium phosphate nano-clusters [19], or between polymeric casein aggregates and casein-calcium phosphate aggregates [20]. The structure of the casein micelle is still elusive, but the “hairy” layers of κ-casein on the surface of the micellar structure are generally accepted [21,22]. The casein micelle is extremely stable in heat treatment, due to the κ-casein layers [12]. κ-casein is the only glycosylated casein with oligosaccharides attached to throline residues in the C-terminal, and the only calcium insensitive casein [23]. The C-terminal, due to the increased hydrophilicity, is the “hairy” structure that extends into the serum solution, which contributes a stabilizing force [23,24]. During the production of cheese or rennet casein, chymosin is added to cut off the κ-casein tail, and the casein collapses to form a weak gel due to the loss of the inter-micellar repulsion force [22]. That is why sweet whey protein (the whey produced from cheese making) contains a significant amount of Glycomacropeptide (GMP), which is one third of κ-casein (residues 106–169) and is present in whey protein products [25]. The casein micelle is made up of 94% casein protein and 6% CCP [26]. CCP stabilizes the casein micelle by crosslinking casein aggregates [27,28,29,30]. During acidification, such as using acid casein, in yogurt making, or in the natural milk-souring process, CCP is dissolved when the pH drops and causes a gradual dissolution of micellar integrity [26,28], and then casein starts to aggregate or precipitate.

The casein micelle is a very complex polymer aggregate, and interacts via hydrophobic and electrostatic interactions and calcium bridging [31]. A typical casein micelle contains about 10,000 casein molecules [11]. In the past, bovine casein molecule were usually called random coil protein because of the high content of proline residues, which prevents the formation of α-helix or β-sheet structures [32,33]. Casein has extreme heat stability and shows no evidence of denaturation to a more disordered structure [34]. The term “rheomorphic” was also suggested for this type of protein [35]. Caseins, considered rheomorphic proteins, have been adopted by many researchers [36,37,38]. According to de Kruif and Holt’s definition, a rhomorphic protein is “one with an open conformation and therefore has a considerable degree of side chain, and possibly also backbone, conformational flexibility” [5].

### 2.2. Whey Proteins

Whey is the liquid that remains after milk is curdled or strained. In the dairy industry, whey refers to the co-products during the process of cheese, casein, or yogurt manufacturing. Cheese whey is currently the most abundant commercially available whey product. Whey proteins are a group of soluble proteins that are present in the milk serum phase at a pH of 4.6.

β-Lg is the dominant protein in whey, which accounts for about 50% of total whey proteins. The molecule is comprised of 15% α-helix (residues 129–143, 65–76 ), 50% β-sheet (residues 1–6, 11–16, 39–45, 80–85, 92–96, 101–107, 117–123, and 145–151), and 15%–20% reverse turn (residues 7–10, 49–52, 61–64, 88–91, and 112–115) [39]. It has a very organized secondary structure, which is described as a “cup” or “calyx” shaped three-dimensional structure [40,41]. Each monomer has one thiol group (residue 121), and two disulphide bonds (residues 160–66 and 106–119) [42,43,44]. The presence of the thiol group and the disulphide bond gives β-Lg an excellent gelation property via a thiol-disulphide interchange under heat treatment [45]. β-Lg can exist in the form of a monomer, dimer, or octamer via the disulphide bonds, depending on pH, temperature, and ion strength [46,47]. It exists as a dimer in neutral pH aqueous solutions [47].

α-La is a globular protein with a two-lube structure [48]. It has 123 amino acid residues and contains four disulphide bonds, but has no thiol group [8,49]. The absence of the thiol group gives α-La a better heat stability than β-Lg. Unlike the thermo-gelling property of β-Lg, α-La forms a molten globule state under mild denaturing conditions [7,50].

BSA is the third major proteins in whey. It is found in both mammal blood and milk. The molecular structure of BSA is much large than that of β-Lg or α-La; it has approximately 580 residues. BSA has both thiol groups and disulphide bonds [51].

## 3. Milk Protein Products

### 3.1. Casein Products

Casein products include rennet casein, acid casein, and caseinate [52]. Production starts with skim milk, and then casein is separated from milk serum by adding rennet (mimicking cheese making), acid (mimicking natural milk souring) [53], or using membrane filtration [54]. Rennet is an enzyme complex that comes from the stomach of ruminant mammals. Chymosin is the key functional enzyme, which hydrolyzes the bond between methionine and phenyalanine in κ-casein; thus, the casein coagulated into three-dimensional networks. The gel is cut, stirred, drained, and washed before being dried into a powder [55]. Casein can be precipitated at the pH of its isoelectric point, which is thought to be 4.6. The acidification can be achieved using mineral acids, e.g., hydrochloric acid or sulphuric acid, or lactic acid produced by microbiological fermentation [56]. Caseinates (sodium, potassium, or calcium caseinate) can be obtained by adding alkalis (sodium hydroxide, potassium hydroxide, or calcium hydroxide) to acid casein.

### 3.2. Whey Protein Products

Whey products were initially viewed as a waste byproduct of cheese for thousands of years of cheese history [57]. Whey can be basically categorized into acid whey and sweet whey [58]. Sweet whey is the co-product of rennet cheese production, and the pH is usually around 6.0 or higher. Acid whey comes from cottage cheese or Greek yogurt, and is formed where milk is acidified. Acid whey has a pH below 5.0 and has a high lactic acid content; the direct processing of acid whey is limited [59], and it is usually considered as the problematic stream. However, the recent Greek yogurt boom in the USA has driven studies on acid whey utilization and processing [60,61,62], and acid whey is presently repeating the success of sweet whey utilization.

Liquid sweet whey can be spray-dried into a powder. Sweet whey powder contains mostly lactose (~80%) and protein (~10%). Membrane technology has been widely used for further processing of whey products [63]. Whey protein concentrate (WPC) with a protein level up to 80% (e.g., WPC34 and WPC80) can be produced using ultrafiltration (UF) [64]. Whey protein isolate (WPI) with a protein content around 90%, can be achieved by combining with microfiltration (MF) in order to remove excess fat [65]. The ash level can be further reduced by nano-filtration, ion-exchange, or resin to produce deminilized whey protein products [66,67].

### 3.3. Membrane Filtered Milk Protein

Membrane filtration technology is able to separate milk protein from the serum phase, based on the different sizes of molecules. Different membrane pore sizes result in different milk protein products. Milk protein concentrate or isolate (MPC or MPI) is produced by ultrafiltration (UF) Both casein and whey proteins are retained by the membrane, but lactose and minerals permeate [68]. Casein and whey protein can be split by a microfiltration (MF) membrane, with casein being retained while whey protein, along with lactose and minerals, can pass through the membrane, which results in micellar casein products [69]. The whey stream can go through a process similar to the cheese whey process to produce so-called milk serum protein concentrate and isolates [70].

## 4. Milk Protein Polymerization

**Figure 1:**
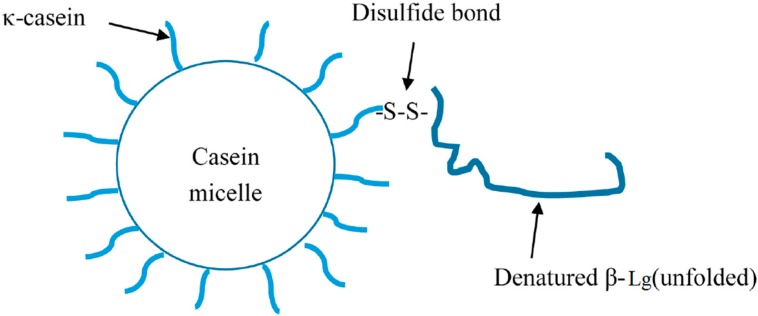
Heat-induced casein and β-Lg interactions.

**Figure 2:**
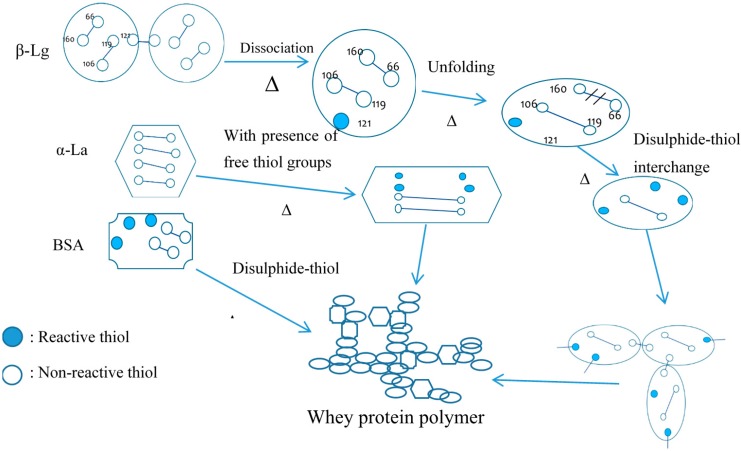
Whey protein polymerization.

Protein polymerization is essential for milk protein to be used as an adhesive. The casein micelle itself is a heterogeneous polymer complex, made of different casein molecules. Additionally, casein also interacts with whey protein to form casein–whey aggregates. The thiol group on the protruding κ-casein tail can interact with β-Lg via thiol-disulphide interchange under heat treatment [71,72,73]. On the other hand, β-Lg can interact with α-La and BSA via the disulfide bonds [74,75]. Thus, a polymer complex of casein and whey protein can be formed under heat treatment [76]. Figure 1 shows the interactions between casein and β-Lg; Figure 2 shows the protein interactions of whey protein molecules.

**Figure 3:**
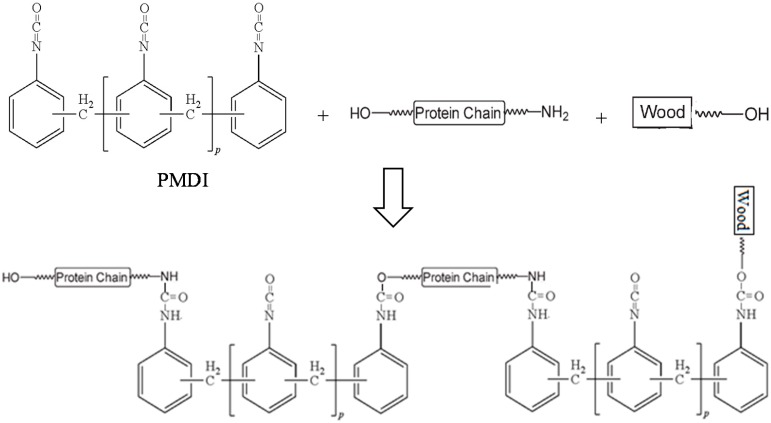
Protein crosslinked by polymeric methylene bisphenyl diisocyanate (PMDI) and the adhesion mechanisms for wood adhesive.

**Figure 4:**
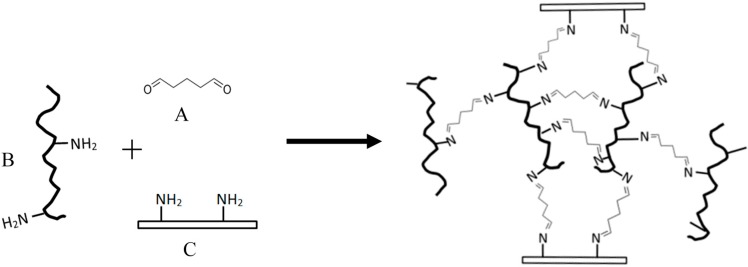
Protein crosslinked by glutaraldehyde and the adhesion mechanism of biological glue. (**A**) glutaraldehyde; (**B**) protein polymer molecules; (**C**) tissue protein.

In order to obtain a strong adhesive strength or fast setting properties, protein molecules may need to be crosslinked. Protein crosslinking can be achieved by physical (irradiation, heat treatment, etc.) [77], enzymatic [78,79], or chemical (adding crosslinker) methods [80]. The strength of a protein network is essential to the bonding strength. Most steps in preparing protein polymer, such as heat treatment, pH adjustment, and the addition of other ingredients, focus on how to increase protein network strength. Mixing protein polymers with chemical crosslinkers is one of the most common methods to cure protein adhesives. Figure 3 and Figure 4 demonstrate examples of how protein is crosslinked, and also the adhesion mechanisms of wood and tissue adhesives. Thermal crosslinking is widely used in plywood manufacturing. By applying both heat and pressure, protein polymers, especially heat-sensitive proteins, are denatured and pressed and are able to form a very firm adhesive film. A hot press is usually used for plywood manufacturing in order to increase the bonding strength [81].

## 5. Milk-Protein-Based Adhesives Applications

### 5.1. Casein-Based Adhesives

The use of casein as glue products can be dated back to ancient Egypt [82]. The first US patent on using casein products can be traced back to the late-19th century [83,84]. The early casein adhesive preparation usually started with sour milk or cheese with decanting whey to remove fat and water, and then it was subjected to a boiling process to further remove water and to denature protein [83,84]. Other ingredients, such like alkali, limewater, or urea, were always added [83,84,85,86]. The casein micelle is very sensitive to environmental changes, such as pH and ion strength [5,87]. Acid precipitation is the easiest way to separate casein from milk, which was widely used in the early days. With the washed and concentrated acid casein aggregates, alkali is usually added to solubilize the casein into viscous, paste-like glue. Alkali pH is able to reassemble the micelle structure of casein that has been collapsed by acidification [87,88]. Urea and ammonia were other common ingredients for casein adhesives in the early days [85,89,90] to lower the viscosity by decreasing the H-bonds [91,92]. During an acid rinse, CCPs are solubilized into the serum phase; thus, the micelle structure collapses, and, combined with a heat process, casein can form a thick, insoluble, adhesive slurry, which resembles white glue. In most cases, casein glues are available with a casein powder and an alkali, which are ready to be mixed in water prior to use [82]. Many of the casein glues are generally no longer used due to the advent of synthetic adhesive polymers; however, some are still in use [82]. In general, casein adhesive is easy to process and provides good bond strength [82].

Casein-based adhesives are still used as a bottle label adhesive [82]. Bottle-labeling adhesive for beer and soda bottles usually required ice-proof or water resistant properties, but also needed to be easily washed off, since bottles may need to be returnable [93]. Acid-precipitated casein, combined with metallic salts as crosslinkers, has been used for this purpose for about a century [86,94]. Casein-based wood glue was very common, due to its excellent water resistance, until synthetic resins were invented [10,90]. The commercial products are usually sold as a dry powder, ready to be mixed with water, right before use, because casein wood glue has a very short pot life. Some casein wood adhesives are still in use today. One current commercial example is “Casein glue-N” from E Min Ta An Casein Co. Ltd. (Xinjiang, China), which is used for furniture crossbanding and for beer labels.

### 5.2. Whey-Protein-Based Adhesives

A globular structure is not ideal for adhesive applications; therefore, treatments (such as heat and solution polarity change) that denature the protein must be applied to globular proteins for adhesive use [95]. Unlike the long history of casein adhesives, the use of whey protein for adhesives is relatively new. Tschabold patented an adhesive from whey in 1953, which used condensed whey [96]. After that, there was very little to be found in the literature until recent years.

WPI has been used as a wood adhesive for both interior and exterior applications [97,98,99,100,101]. Similar to casein wood adhesive, whey protein wood adhesive is also a ready-to-mix adhesive containing a whey polymer aqueous solution and a crosslinker. The process usually starts with a high concentration of WPI solution (20%–40%) polymerized at 60 °C or higher, and is then combined with a synthetic co-polymer, such as polyvinyl acetate (PVAC) and polyvinyl alcohol (PVA). The polymer suspension is ready to be mixed with a crosslinker, such as phenol-formaldehyde oligomer or polymeric methylene bisphenyl diisocyanate. Due to the thermo-setting properties of whey protein, desirable bonding strength can also be obtained by hot pressing the whey protein polymer suspension without crosslinker at 120–140 °C [100].

Office or paper glue is another area where whey protein can be applied due to safety concerns [102]. Current commercial paper glues are commonly PVAC- and PVA-based. Polymerized whey protein could be able to provide a desirable bonding strength, but the control of viscosity during storage, to make the shelf life stable, is a challenge [103]. Polymerized whey protein suspension can be combined with PVAC or PVA to obtain a good bonding strength, but the glue suspension is likely to gel during the shelf life [103]; however, polymerized whey protein with polyvinyl pyrollidone (PVP) as a co-binder has demonstrated very good bonding strength and a stable shelf life (at least two years) [103]. Carbohydrates (like sucrose) could be used with the polymerized whey protein suspension in order to maintain the viscosity constant during storage [104]. Whey protein with PVP can also be used for glue stick applications [102].

β-Lg, α-La, and BSA are all rich in lysyl residues, which have ε-amino groups [51,105,106]. The ε-amino group is very active and can react with crosslinkers, such as glutaraldehyde (GTA). Globular protein and GTA adhesive has been commercially available for use as surgical glue, under the brand BioGlue^®^. Tissue adhesive is a new alternate device for sutures, which could reduce physical pain, prevent fluid leakage, an to shorten operation times [107,108]. BioGlue^®^ is an FDA approved tissue adhesive, which contains BSA as a protein polymer and GTA as a crosslinker. BSA is found in both bovine blood and milk. Extensive in vivo evaluation of BioGlue^®^ has been carried over the past 10 years [109,110,111,112,113,114]. The reaction mechanism of protein and GTA for use as tissue adhesive is depicted in Figure 4. GTA can bridge the protein polymer and the tissue cells via reaction with the free amino groups of the protein polymers and tissue cell proteins [108].

## 6. Summary

Synthetic petroleum-based resin will still dominate the adhesive industry due to its low cost and excellent performance. However, as the rising concerns of environment pollution, human health, and inert waste disposal, with respect to the use or production of synthetic adhesive polymers, environmentally safe bio-resourced and renewable polymers still have good opportunities of use [115], such as for paper glue for office/school/home use or in food packaging, wood adhesives, and biological glues, where safety and other factors are more important than cost. Therefore, milk protein or other natural polymer-based adhesives will have a niche in the market.

## Figures and Tables

**Table 1:** Major proteins in bovine milk.

Proteins	Content, g/L
Casein	25
α-Casein	12
β-Casein	9
κ-Casein	3.25
Minor constituents	0.75
Whey Protein	5.4
β-lactoglobulin	2.70
α-lactoalbumin	1.20
Serum albumin	0.65
Minor constituents	0.85
